# Origin of O_2_ Generation in Sulfide‐Based All‐Solid‐State Batteries and its Impact on High Energy Density

**DOI:** 10.1002/advs.202402528

**Published:** 2024-07-08

**Authors:** Keisuke Yoshikawa, Takeshi Kato, Yasuhiro Suzuki, Akihiro Shiota, Tsuyoshi Ohnishi, Koji Amezawa, Aiko Nakao, Takeshi Yajima, Yasutoshi Iriyama

**Affiliations:** ^1^ Department of Material Design Innovation Engineering Graduate School of Engineering Nagoya University Furo‐cho, Chikusa‐ku Nagoya Aichi 464‐8603 Japan; ^2^ Consortium for Lithium Ion Battery Technology and Evaluation Center (LIBTEC) 1‐8‐31 Midorigaoka Ikeda Osaka 563‐8577 Japan; ^3^ Center for Green Research on Energy and Environmental Materials National Institute for Materials Science (NIMS) 1‐1 Namiki Tsukuba Ibaraki 305‐0044 Japan; ^4^ Institute of Multidisciplinary Research for Advanced Materials Tohoku University 2‐1‐1, Katahira, Aoba‐Ku Sendai Miyagi 980‐8577 Japan

**Keywords:** all‐solid‐state battery, electrochemistry, energy conversion, interfaces, mass spectrometry

## Abstract

The cathode surface of sulfide‐based all‐solid‐state batteries (SBs) is commonly coated with amorphous‐LiNbO_3_ in order to stabilize charge–discharge reactions. However, high‐voltage charging diminishes the advantages, which is caused by problems with the amorphous‐LiNbO_3_ coating layer. This study has investigated the degradation of amorphous‐LiNbO_3_ coating layer directly during the high‐voltage charging of SBs. O_2_ generation via Li extraction from the amorphous‐LiNbO_3_ coating layer is observed using electrochemical gas analysis and electrochemical X‐ray photoelectron spectroscopy. This O_2_ leads to the formation of an oxidative solid electrolyte (SE) around the coating layer and degrades the battery performance. On the other hand, elemental substitution (i.e., amorphous‐LiNb*_x_* P_1‐_ *_x_* O_3_) reduces O_2_ release, leading to stable high‐voltage charge–discharge reactions of SBs. The results have emphasized that the suppression of O_2_ generation is a key factor in improving the energy density of SBs.

## Graphical abstract

O_2_ generation happens around the surface of an amorphous‐LiNbO_3_ coating layer on the electrode during high‐voltage charging of sulfide‐based solid‐state batteries (SBs) due to Li extraction from the coating layer, and an amorphous‐LiNb*_x_* P_1‐_ *_x_* O_3_ coating layer suppresses O_2_ generation, contributing to the development of high‐energy‐density SBs.

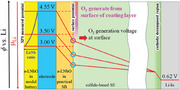

## 1 Introduction

Sulfide‐based all‐solid‐state batteries (SBs) are expected as advanced power sources for electric vehicles (EVs). This is because sulfide‐based solid electrolytes (SEs) are highly Li^+^ conductive materials^[^ ^1^ ^]^ and are ductile,^[^ ^2^ ^]^ which are beneficial for fast charge–discharge reactions in the SBs of EVs and large‐scale battery manufacturing processes. However, SEs have a narrow potential window^[^ ^3^ ^]^ and they are highly reactive with cathode materials, leading to a resistive interface.^[^ ^4^ ^]^ To overcome these problems, a coating layer, commonly amorphous‐LiNbO_3_ (a‐LNbO, ≈10 nm in thickness), is formed on the cathode surface and several issues have been examined.^[^ ^5^ ^]^

A practical method for improving the energy density of SBs is to increase their charging voltages. However, high‐voltage charging degrades SBs and several types of degradations can occur inside SBs, such as deterioration of the cathode structure,^[^ ^6^ ^]^ degradation of the coating layer,^[^ ^3^ ^]^ and oxidation of the SEs around the coating layer.^[^ ^7^ ^]^ A typical cathode material, LiNi*_x_* Co*_y_* Mn_1−_ *_x_*_−_ *_y_* O_2_ (NCM), releases O_2_ during high‐voltage charging (≈4.55 V), and the layered structure of NCM irreversibly transforms into a resistive spinel or rock‐salt structure.^[^ ^6^ ^]^ Gas analysis has been used to detect O_2_ release during the SB charging process, as well as CO_2_ (due to the decomposition of Li_2_CO_3_ on the NCM surface) and SO_2_ (due to side reactions with SEs).^[^ ^8^ ^]^ Coating layers protect NCM and reduce O_2_ release from NCM.^[^ ^6^, ^8^ ^]^ However, coating layers also degrade at high‐voltage charging and become alternative degradation sources.^[^ ^7^ ^]^ In the case of the a‐LNbO coating layer, the following decomposition schemes have been proposed indirectly based on X‐ray absorption fine structure (XAFS) analysis of Nb L_3_‐edge local structure variations at high voltages:^[^ ^7d^ ^]^

(1) $$\mathrm{LiNb}O_{3}\to Li_{1-2x}\mathrm{Nb}O_{3-x}+xLi_{2}O$$

(2) $$Li_{2}O\;\;\to 0.5O_{2}+2Li^{+}+2e^{-}$$

In this study, we have investigated the gas generation from SBs with and without coating layers on the NCM. We demonstrate that a larger amount of O_2_ is generated from the a‐LNbO coating layer during high‐voltage charging. Additionally, Li extraction from the a‐LNbO coating layer is confirmed using electrochemical X‐ray photoelectron spectroscopy (XPS) analysis. These results directly support the forementioned degradation schemes. Based on these results, we propose that the design of coating layers that suppress O_2_ generation is a key factor in stabilizing the high‐voltage charging of SBs. In fact, we will show that an amorphous‐LiNb*_x_* P_1‐_ *_x_* O_3_ (a‐LNbPO) coating layer, which is known to improve the high‐voltage stability of SBs,^[^ ^9^ ^]^ generates less O_2_.

## 2 Results

### 2.1 Gas Analysis Using Practical SBs

**Figure 1:**
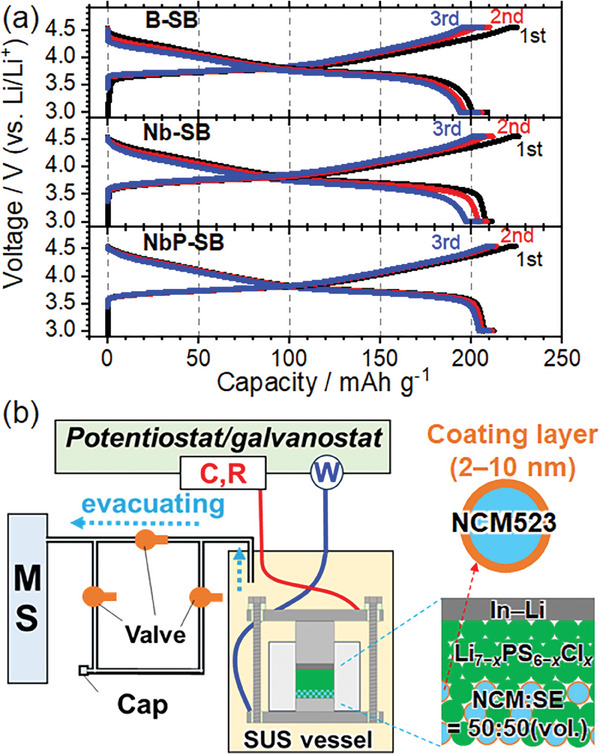
a) Charge–discharge curves of B‐SB (upper), Nb‐SB (middle) and NbP‐SB (lower) from 1st to 3rd cycle (3.00–4.55 V, 60 °C, *I* = 200 µA cm^−2^). b) Schematic image of gas analysis system. SBs are sealed in SUS vessel, which are stored at 60 °C and connected to mass spectrometer (MS) and potentiostat‐galvanostat.

The SBs used for gas analysis comprise LiNi_0.5_Co_0.2_Mn_0.3_O_2_ (NCM523) particles as the cathode, Li_7−_ *_x_* PS_6−_ *_x_* Cl*_x_* (*x* ≈1, conductivity ≈2 × 10^−3^ S cm^−1^) as the SE, and In–Li alloy (0.62 V vs Li/Li^+[^ ^10^ ^]^) as the anode (FigureS1, Supporting Information). In this study, three types of SBs with a‐LNbO‐coated NCM523 (Nb‐SB), a‐LNbPO‐coated NCM523 (NbP‐SB), or bare NCM523 (B‐SB) were assembled according to previous reports, where the thickness of each coating layer was 2–10 nm.^[^ ^7^, ^9^ ^]^ **Figure** **1**a shows the charge–discharge curves of the SBs at 60 °C. The delivered capacities and Coulombic efficiencies were in the order of NbP‐SB > Nb‐SB > B‐SB, confirming that coating layers improve the battery performance as shown in our previous reports.^[^ ^7^, ^9^ ^]^ Also, we have preliminary checked that a‐LNbPO coating layer has improved charge–discharge reactions of an SB when compared with a‐LNbO coated one using NCM523‐graphite laminate cell operating at 60 °C for 300 cycles between 3.00 and 4.35 V (≈4.4 V vs Li/Li^+^) (Figure S2, Supporting Information). The SBs were sealed in a stainless use steel vessel. The vessel was placed in an incubator (60 °C) and was connected to a gas analyzer and potentiostat–galvanostat. Gas analysis was performed under vacuum with constant voltages applied to the SBs (Figure 1b; Figure S3, Supporting Information).

**Figure 2:**
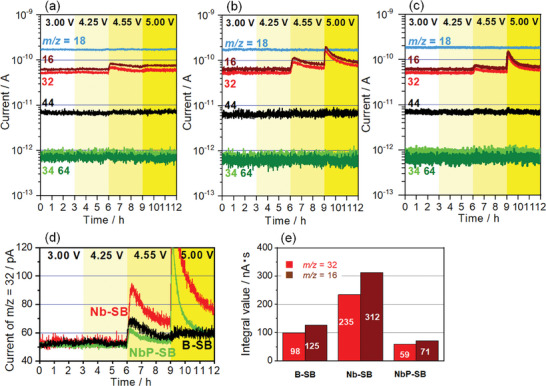
Time‐dependent ion currents for *m/z* = 16 (brown), 18 (light blue), 32 (red), 34 (light green), 44 (black), and 64 (dark green) of a) charged B‐SB b) charged Nb‐SB, and c) charged NbP‐SB. d) Comparison of the current for *m/z* = 32 from B‐SB (black), Nb‐SB (red) and NbP‐SB (light green). e) Integral amount of *m/z* = 16 (brown), 32 (red) at 4.55 V for 3 h from B‐SB, Nb‐SB and NbP‐SB.

**Figure** **2**a–c summarizes the gas analysis results for the B‐SB, Nb‐SB, and NbP‐SB, respectively, where the voltages of the SBs were increased every 3 h to 3.00, 4.25, 4.55, and 5.00 V (vs Li/Li^+^). The mass‐to‐charge ratios of the ions (*m/z*) (16 (O), 18 (H_2_O), 32 (O_2_ or S), 34 (H_2_S), 44 (CO_2_), and 64 (SO_2_)) were selected based on previous reports and gas leaks from air.^[^ ^8^, ^11^ ^]^ An empty vessel without SB guaranteed constant ion currents at any *m/z* for 12 h in our system (Figure S3b, Supporting Information). At 3.00 and 4.25 V, the ion currents of the selected *m/z* values were nearly consistent with those of the empty vessel. At 4.55 V, the ion current from *m/z* = 16 and 32 increased and decayed with time. At 5.00 V, the ion current increased again only for *m/z* = 16 and 32, with the exception of B‐SB. Figure 2d compares the ion currents at *m/z* = 32 for the SBs. The Nb‐SB generated the highest peak current and the NbP‐SB generated the lowest peak current among the SBs at 4.55 V. Figure 2e summarizes the integral amount of *m/z* = 16 and 32 at 4.55 V calculated from the peak area for 3 h in Figure 2d. Each integral amount for the Nb‐SB was 2.4–2.5 times larger than that for the B‐SB, whereas that of the NbP‐SB was 0.6 times smaller than that of the B‐SB. Another possible assignment of *m/z* = 32 (S) was examined by using S poisoning of Si/Ti/Pt substrates (Figure S4, Supporting Information). Although a small amount of S was continuously generated probably because of the chemical reaction between the SE and the leaked H_2_O, the generated S was not voltage‐dependent. Thus, S was detected as part of the baseline current at *m/z* = 32 in Figure 2a–c. The voltage‐dependent peak current at *m/z* = 32 was assigned to O_2_ providing a fragment with *m/z* = 16 (O). These results demonstrate that O_2_ is generated not only from NCM523^[^ ^6^ ^]^ (Figure 2a) but also from the a‐LNbO coating layer (Figure 2b) during high‐voltage charging. Because NCM523 maintains a layered structure even after the charge–discharge cycles at 4.55 V,^[^ ^7b^ ^]^ O_2_ is mainly generated from the a‐LNbO coating layer. In addition, the a‐LNbPO coating layer further reduced the O_2_ release. These results directly support Equation (2), based on O_2_ generation. The other *m/z* ratios (1–100) of the SBs did not exhibit any voltage dependence (Figure S5, Supporting Information).

### 2.2 XPS Analysis Using Model Batteries

**Figure 3:**
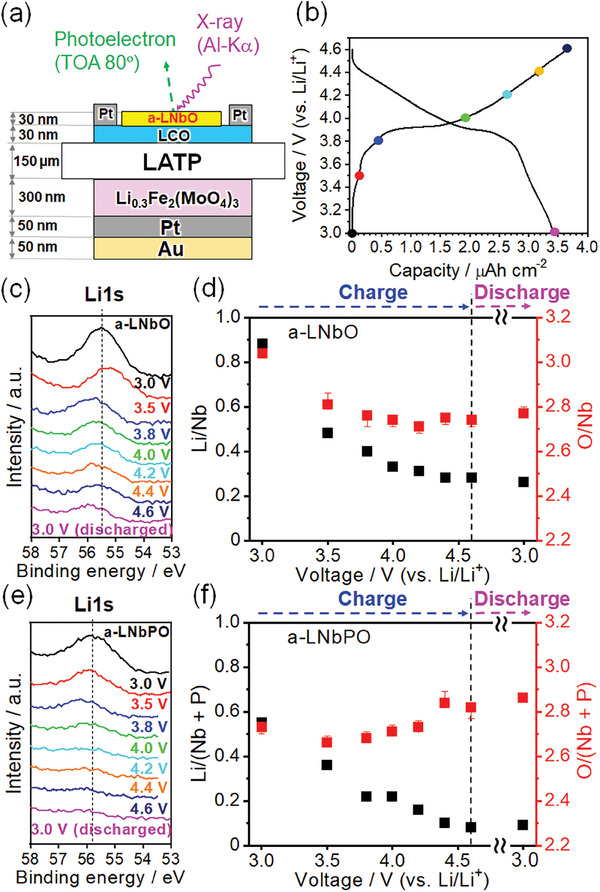
a) Schematic image and b) the initial charge–discharge curve (3.0–4.6 V vs Li/Li^+^, *I* = 1.0 µA cm^−2^, room temperature) of FMO/LATP/LCO/a‐LNbO model battery for electrochemical XPS measurements. Voltage dependencies of c) Li1s XPS spectra and d) atomic ratios of Li/Nb (black) and O/Nb (red) on the a‐LNbO film estimated by XPS (SI‐8). Voltage dependencies of e) Li1s XPS spectra and f) atomic ratios of Li/(Nb + P) (black) and O/(Nb + P) (red) on the a‐LNbPO film estimated by XPS (Figure S10, Supporting Information).

The amount of Li in the a‐LNbO coating layer during the charging process was examined using electrochemical XPS to validate Equation (1). In this study, oxide‐based solid‐state batteries with LiCoO_2_ (LCO) cathode films and flat‐textured Li^+^‐conductive solid electrolyte sheet (Li_1.3_Al_0.3_Ti_1.7_(PO_4_)_3_ (LATP)) were constructed in order to investigate variations in the element ratios with voltage around the electrode/coating layer interface. An a‐LNbO film (30 nm thick) was formed on the LCO films as a model coating layer using pulsed laser deposition (PLD), ensuring no exposure of the sample to air. Preliminary tests confirmed that the a‐LNbO film functioned effectively as a coating layer in the SB (SI‐6). **Figure** **3**a shows a schematic of a model solid‐state battery, (Fe_2_(MoO_4_)_3_ (FMO)/LATP/LCO). FMO operates with a potential plateau at 3.0 V (vs Li/Li^+^) and works as a reference and counter electrode on LATP.^[^ ^12^ ^]^ The cathode side of the model battery was equipped with a Pt current collector only around the edges of the LCO film (Figure S7, Supporting Information). Figure 3b shows the charge–discharge curve of the battery, with the voltage converted to Li/Li^+^. During the charge–discharge reactions, the voltage was held constant for the XPS measurements. Co2p_3/2_, Li1s, Nb3d, and O1s XPS spectra were measured at the same position on the a‐LNbO film (SI‐8). Co2p_3/2_ was not detected at any voltages (Figure S8a, Supporting Information), confirming that Li1s was analyzed only in the a‐LNbO film. Figure 3c shows the voltage dependence of the Li1s peak. The intensity of the Li1s peak decreased with increasing the voltage. These results directly indicate that Li is extracted from the a‐LNbO film during charging, supporting Equation (1) based on Li extraction. Figure 3d summarizes the variations found in both the Li/Nb and O/Nb atomic ratios during the initial charge–discharge reaction. At 3.0 V, the Li/Nb and O/Nb ratios were 0.88 and 3.04, respectively. At 3.5 V, the Li/Nb ratio decreased to 0.48, and the O/Nb ratio to 2.81, suggesting that the a‐LNbO layer oxidizes with O_2_ release. Above 3.8 V, the Li/Nb ratio further decreased with increasing charging voltages, whereas the O/Nb ratio remained at ≈2.8, indicating that the a‐LNbO film oxidized without O_2_ release. Even after discharging to 3.0 V, these ratios did not return to their initial values, thus implying irreversible decomposition of the a‐LNbO film during the initial charging through Li extraction. Delithiation occurred in the same manner inside the a‐LNbO film until reaching the LCO layer (Figure S8e,f, Supporting Information). The valence band maximum (VBM) of the a‐LNbO film was 5.8 eV (Figure S9, Supporting Information), which is 3.3 eV lower than the work function of Li metal (2.5 eV).^[^ ^13^ ^]^ Therefore, the oxidative decomposition of a‐LNbO below 3.5 V is reasonably explained also by the electronic properties of the a‐LNbO film.

## 3 Discussion

The XPS and gas analyses supported Equations (1) and (**2**), except for the phase separation of Li_2_O and its subsequent decomposition. The amount of Li extracted (ΔLi/Nb = 0.40) at 3.5 V is almost double that of the removed amount of O (ΔO/Nb = 0.23). In contrast, Li_2_O is not separately detected in the XPS analyses, and 3.5 V is a sufficient voltage to decompose Li_2_O.^[^ ^14^ ^]^ Hence, it is reasonable to expect that O_2_ is released simultaneously with the Li extraction at lower voltages.

**Figure 4:**
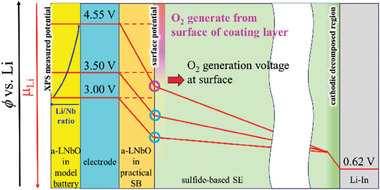
Schematic image of potential profiles in an SB with a‐LNbO coating layer.

The surface of the a‐LNbO film shown in Figure 3a does not contact with the SE, but it still decreases the Li/Nb ratios during charging. Thus, we expect the Li chemical potential (μL_i_) inside the coating layer to be almost uniform and closely aligned with that of LCO, where μL_i_ in LCO decreases with increasing charging voltage (*ϕ*). On the other hand, in SBs, the a‐LNbO coating layer contacts with both NCM523 and SE, and then μL_i_ in a‐LNbO film must be aligned with these materials at each boundary as shown in **Figure** **4**.^[^ ^3a^ ^]^ Here, Li‐deficient a‐LNbO has to be formed at NCM523 boundary according to XPS analyses, while Li‐rich a‐LNbO will be formed alternatively at the SE boundary through phase separation or diffusion of both Li^+^ and O^2−^ toward the SE side.^[^ ^15^ ^]^ Here, the XPS analyses detect O_2_ generation at 3.5 V, while gas analyses detect O_2_ generation over 4.25 V. The anodic potential window calculated for crystalline LiNbO_3_ and other lithium niobium oxides lies 3.0–4.0 V,^[^ ^3^ ^]^ and then XPS results are consistent with the calculated potential window while the gas analyses results exceed it. Thus, we expect that O_2_ is released around the surface of the a‐LNbO film once the SE boundary voltage (surface potential) of the a‐LNbO film exceeds the threshold voltage for O_2_ generation. Anodic decomposition of the SE by electron leakage through a‐LNbO is likely because of the very narrow potential window of the SE (≈0.3 V).^[^ ^3^, ^16^ ^]^ Such a decomposed SE reacts with the generated O_2_ and increases the amount of oxidative SE (PO*_x_* etc.) around a‐LNbO, which is strongly related to the capacity retention of the SBs operating at high voltage.^[^ ^7^ ^]^ In the case of thicker a‐LNbO, electron leakage through the a‐LNbO is suppressed, and then O_2_ generation will be further reduced.^[^ ^16^ ^]^ Also, if the electronic conductivity of the a‐LNbO film does not change even after degradation, thicker a‐LNbO films can reduce the surface potential and subsequently reduce O_2_ generation at a given charge voltage.^[^ ^17^ ^]^ In fact, a thicker and more uniform a‐LNbO coating layer improves capacity fading for the long‐term cycling performance of SBs.^[^ ^16^ ^]^

Gas analyses clarified that the amount of O_2_ release was significantly reduced by applying a‐LNbPO instead of a‐LNbO as a coating layer. Electrochemical XPS measurements of the a‐LNbPO film (30 nm thick, P/Nb≈0.5) were also conducted similarly to those of the a‐LNbO film in order to elucidate the mechanism (Figure S10, Supporting Information). The a‐LNbPO film also worked as a coating layer in an SB (Figure S6, Supporting Information). Figure 3e illustrates the voltage dependence for the Li1s peak from the a‐LNbPO film. The intensity of the Li1s peak progressively decreased during the charging process. Figure 3f summarizes the variations in both the Li/(Nb + P) and O/(Nb + P) atomic ratios during the initial charge–discharge reaction. At 3.0 V, the Li/(Nb + P) and O/(Nb + P) ratios were 0.55 and 2.73, respectively. At 3.5 V, the Li/(Nb + P) ratio decreased to 0.36, whereas the O/(Nb + P) ratio decreased slightly to 2.66. Above 3.8 V, the Li/(Nb + P) ratio decreased gradually with an increasing charging voltage, whereas the O/(Nb + P) ratio at 3.5–4.3 V remained within the error‐bar scale at 3.0 V and slightly increased to 2.84 over 4.4 V. Although the chemical shifts of these elements during the charging reaction are not easy to discuss due to the zig‐zag peak energy shift, the O1s of a‐LNbPO appeared unique shoulder peak at a higher binding energy with an increasing charging voltage (Figure S10c, Supporting Information). We predict that this shoulder peak is assigned to peroxide‐like O^−^ formation.^[^ ^18^ ^]^ Assuming that both Nb and P are pentavalent and that both [NbO_6_] octahedral and [PO_4_] tetrahedral units are connected only by corner‐sharing, the ideal O/(Nb + P) ratio is 3, which is close to the experimental value (2.73). During the charging process, a‐LNbPO may oxidize lattice‐O^2−^ to O^−^, and O_2_ generation may be suppressed probably because most of lattice‐Os are strongly bonded to P. Because the O/(Nb + P) ratio increased slightly above 4.4 V, uncaptured oxygen may be trapped by partially breaking the corner‐sharing bonding. Of course, further analyses are required to clarify the aforementioned mechanism in detail to confirm microscopic guidelines to develop advanced coating materials for high‐energy density SBs. After discharged to 3.0 V, these ratios did not return to their initial values. The remaining O1s shoulder at higher binding energy will be due to the slow recovery of Li to delithiated a‐LNbPO. These results indicate that delithiation of the a‐LNbPO coating layer also occurs, but the reaction primarily proceeds through oxidation of the a‐LNbPO film under the capture of oxygen. Thus, we can conclude that suppressing O_2_ generation from the coating layer is crucial for achieving stable and long‐life SBs operating at high voltages.

## 4 Conclusion

In summary, the a‐LNbO coating layer on the NCM523 electrode decomposes via O_2_ release with Li extraction. This O_2_ leads to an oxidative SE around a‐LNbO and degrades the battery performance. Thus, suppressing O_2_ release from the coating layer is a key strategy for stabilizing the high‐voltage charging of SBs, for which a‐LNbPO is a suitable candidate.

The authors have cited additional references within the Supporting Information.^[^ ^4^, ^7^, ^9^, ^12^, ^13^, ^19^, ^20^, ^21^, ^22^, ^23^, ^24^, ^25^, ^26^, ^27^, ^28^, ^29^, ^30^, ^31^ ^]^

## Conflict of Interest

The authors declare no conflict of interest.

5

## Supporting information

Supporting Information

## Data Availability

The data that support the findings of this study are available from the corresponding author upon reasonable request.
