# Supplementary material for: Origin of O2 Generation in Sulfide‐Based All‐Solid‐State Batteries and its Impact on High Energy Density
Source: Adv Sci (Weinh). 2024 Jul 8;11(34):2402528. doi: 10.1002/advs.202402528 (PMC11425888; doi:10.1002/advs.202402528)
Supplement: Supplementary file 1 — Supporting Information [file ADVS-11-2402528-s001.pdf]

ADVANCED SCIENCE
Open Access

# Supporting Information

for *Adv. Sci.*, DOI 10.1002/advs.202402528

Origin of $O_2$ Generation in Sulfide-Based All-Solid-State Batteries and its Impact on High Energy Density

*Keisuke Yoshikawa, Takeshi Kato, Yasuhiro Suzuki, Akihiro Shiota, Tsuyoshi Ohnishi, Koji Amezawa, Aiko Nakao, Takeshi Yajima and Yasutoshi Iriyama**

Supporting Information

**Origin of $O_2$ Generation in Sulfide-Based All-Solid-State Batteries and Its Impact on High Energy Density**

*Keisuke Yoshikawa, Takeshi Kato, Yasuhiro Suzuki, Akihiro Shiota, Tsuyoshi Ohnishi, Koji Amezawa, Aiko Nakao, Takeshi Yajima, and Yasutoshi Iriyama**

K. Yoshikawa, T. Kato, Y. Suzuki, A. Nakao, Prof. T. Yajima, Prof. Y. Iriyama
Department of Material Design Innovation Engineering, Graduate School of Engineering, Nagoya University
Furo-cho, Chikusa-ku, Nagoya, Aichi, 464-8603, Japan
E-mail: iriyama.yasutoshi@material.nagoya-u.ac.jp

A. Shiota
Consortium for Lithium Ion Battery Technology and Evaluation Center (LIBTEC)
1-8-31 Midorigaoka, Ikeda, Osaka, 563-8577, Japan

Dr. T. Ohnishi
Center for Green Research on Energy and Environmental Materials, National Institute for Materials Science (NIMS)
1-1 Namiki, Tsukuba, Ibaraki, 305-0044, Japan

Prof. K. Amezawa
Institute of Multidisciplinary Research for Advanced Materials, Tohoku University
2-1-1, Katahira, Aoba-Ku, Sendai, Miyagi, 980-8577, Japan

# Table of contents


1. Solid State Battery Cells for Gas Generation Analyses ............................................3
2. Cycle stability tests of a-LNbPO and a-LNbO coated NCM523 ................................4
3. Gas analysis of an empty vessel ....................................................................................5
4. S poisoning test ..............................................................................................................6
5. Voltage dependencies of *m/z* (1–100) from charged SBs ..........................................8
6. Preliminary tests of a-LNbO and a-LNbPO films prepared by PLD as coating layers of SBs ..........................................................................................................11
7. Operations of model SBs for electrochemical XPS analyses ...................................12
8. XPS analyses of an a-LNbO film during charging of a model battery ....................14
9. Electronic properties of a-LNbO and a-LNbPO films prepared by PLD .................16
10. XPS analyses of an a-LNbPO film at charging of a model battery ........................17
11. References ................................................................................................................ 18

# 1. Solid State Battery Cells for Gas Generation Analyses

$LiNi_{0.5}Co_{0.2}Mn_{0.3}O_2$ (NCM523, Sumitomo Metal Mining) was utilized as the cathode active material. The amorphous $LiNbO_3$ (a-LNbO) coating material, having a Li:Nb=1:1 molar ratio, was prepared using a Li–Nb double ethoxide anhydride ethanol solution.[1,2] The amorphous-$LiNb_xP_{1-x}O_3$ (a-LNbPO) coating material was prepared by dissolving $LiNbO_3$ and $LiPO_3$ glass powders in ultrapure water, maintaining a molar ratio of 1:1.[2] Coating on the NCM523 surface was accomplished using a rolling-fluidized coating machine (MP series, Powrex) by spraying these solutions. An argillodite-type sulfide-based solid electrolyte, $Li_{(7-x)}PS_{(6-x)}Cl_x$ (LPSCl, $x$~1, density 1.8 g cm$^{-3}$, conductivity ~2 × 10$^{-3}$ S cm$^{-1}$, Mitsui Mining & Smelting), was selected as the solid electrolyte (SE). Thickness of those coating layers is ca. 2–10 nm. [1,2]

Figure S1 illustrates a schematic of an all-solid-state battery (SB). The fabrication processes of the SBs were consistent with previous reports. [1,2] A mixture of NCM523 powder (either a-LNbO-coated or a-LNbPO-coated or uncoated) and SE powder in a 1:1 volume ratio (71 : 29 weight ratio) was homogenized for 10 minutes using an agate mortar, serving as the raw material for the composite electrode. Initially, the powdered SE (127.3 mg) was filled into a ceramic cylinder (1 cm$^2$ area) and compressed at 98 MPa to form a dense SE layer. Subsequently, the prepared composite electrode material (17.7 mg) was placed on one side of the SE layer and pressed at 588 MPa. Thereafter, an In–Li alloy counter electrode, fabricated by sandwiching a Li foil (200 μm thick, Honjo Metal) between two In foils (100 or 300 μm thick, Nilaco), was mounted on the opposite side of the SE layer and compressed at 98 MPa. The resulting SBs were placed in a holder applying constant constraining pressure in the uniaxial direction. All procedures were conducted in an argon-filled glove box (dew point < −80°C, oxygen concentration < 1 ppm). Preliminary charge/discharge measurements of the SBs were carried out using a potentiostat/galvanostat (VSP-300, Biologic) in a thermostatic chamber (SU-222, Espec) at 60ºC. The results are summarized in Figure 1a.

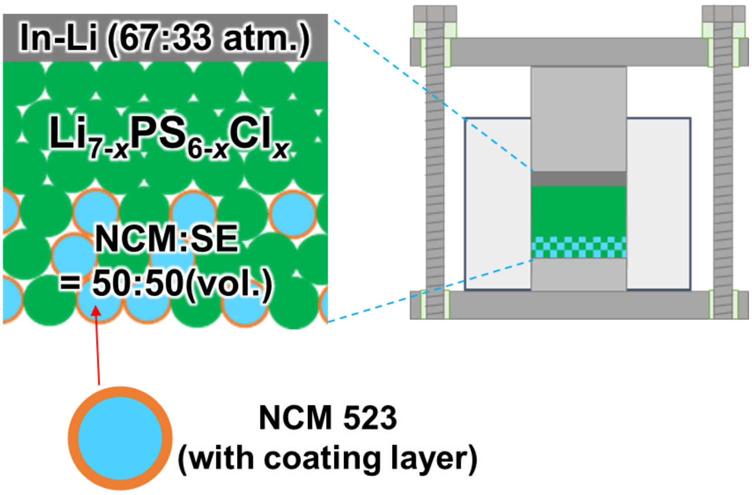


**Figure S1**. Schematic image of a SB and a cell holder.

## 2. Cycle stability tests of a-LNbPO and a-LNbO coated NCM523

The cycle stability of a-LNbPO coated NCM523 electrodes was compared with that of a-LNbO coated ones using laminate cells. The coated NCM523 powders (83 wt%) were mixed with LPSCl, a conductive additive, and a binder in the same manner. The composite electrodes were formed on Al film and pressed (60 μm in thickness). The common negative electrode was a graphite composite electrode and formed in the same manner (80 μm in thickness). These composite electrodes were combined with an LPSCl separator, pressed, and developed into laminate-type cells (4 $cm^2$ in electrode area). Charge-discharge tests of the resultant cells were conducted at 60°C between 3.00 V and 4.35 V (approximately 4.4 V vs. $Li/Li^+$) at a 1C rate for 300 cycles. Figure S2 summarizes the variations of the discharge capacity, revealing that a cell using an a-LNbPO coated NCM523 electrode exhibits better stability than one using a-LNbO coated electrodes.

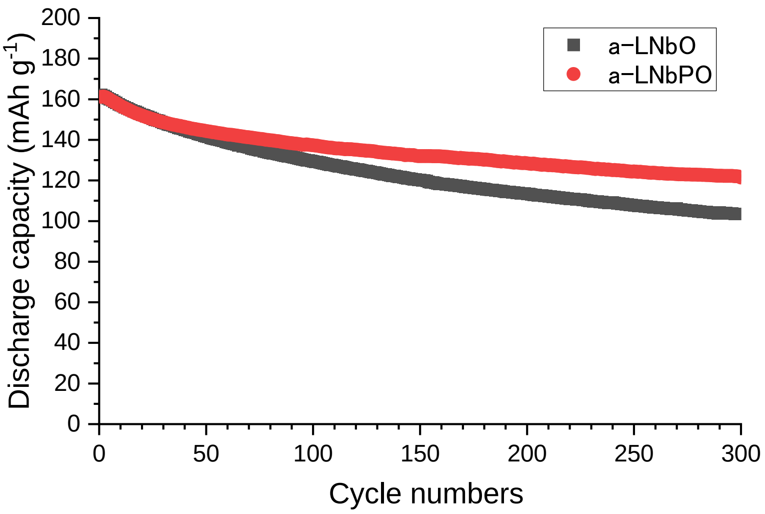


**Figure S2**. The variation in discharge capacity of laminate cells using a-LNbPO coated NCM523 (red) and a-LNbO coated NCM523 (black). The cells were operated at 60°C for 300 cycles between 3.00 V and 4.30 V at a 1C rate.

# 3. Gas analysis of an empty vessel

Figure S3a presents a schematic of the gas analysis system. A stainless steel (SUS) vessel, equipped with electrochemical cables and gas piping, was prepared. A SB was placed inside the SUS vessel in an argon-filled glove box (GB: dew point < −80°C), and then sealed after connecting the electrochemical cables to the SB. The SUS vessel was positioned outside the GB with the valve attached to the vessel (V-V) closed and placed in an incubator set to 60 °C. This vessel was then connected to the gas analysis system. A rotary pump (RP) was installed in this gas line, and after connecting the gas line, the entire system was evacuated with the V-V closed. Once the V-V was opened and the vessel evacuated, both the vessel and gas line were further evacuated using the RP and a turbo molecular pump (TMP) attached to the gas analyzer. This process continued until the pressure measured by the gas analyzer reached a base pressure of 1.5 × $10^{-5}$ Pa.

The electrochemical cable outside the SUS vessel was connected to a potentiostat/galvanostat (SP-150, Bio-logic). Upon reaching the base pressure at 60°C, the SUS vessel was evacuated by RP and TMP, and gas analysis was conducted at 3.00, 4.25, 4.55, and 5.00 V (vs. $Li/Li^+$) every 3 hours while continuing the evacuation of the SUS vessel. For gas analysis, a gas analyzer (M-201GA-CRMY, Canon Anelva) equipped with a quadrupole mass spectrometer (QMA) was used to measure the ion current values for gases with *m/z* = 1–100. A gas analysis was performed using an empty SUS vessel as a reference to provide a baseline measurement. This blank measurement showed constant ion currents for typical *m/z* (16, 18, 32, 34, 44, and 64) values over a 12-hours and stable currents were observed as depicted in Figure S3b.

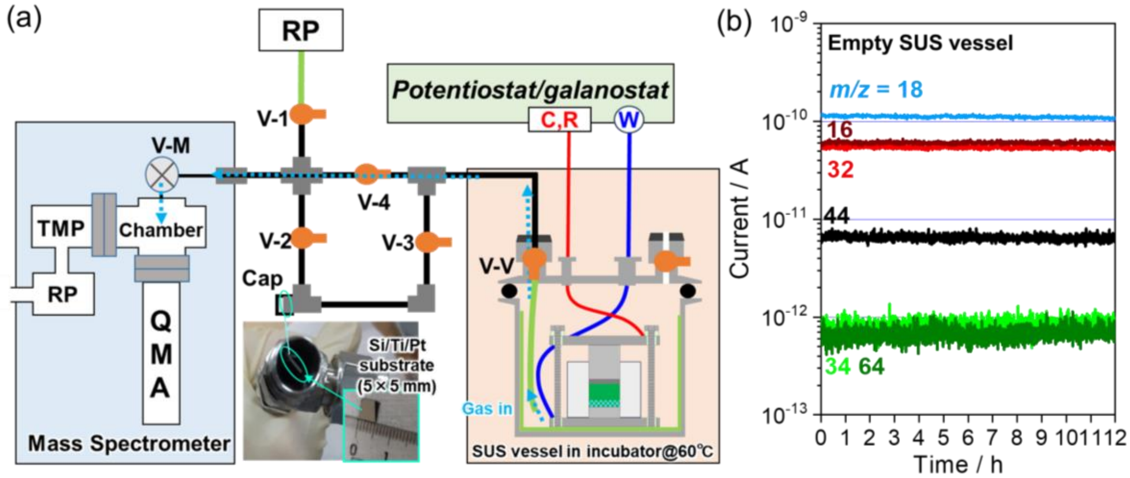


**Figure S3.** (a) Schematic image of the gas analysis system. V: valve, TMP: turbo molecular pump, RP: rotary pump, QMA: quadrupole mass spectrometer, blue line: cable of the working pole (W), red line: cable of the counter and reference electrode (C, R), thick black line: thicker SUS tube, thin black line: thinner SUS tube, yellow-green line: Teflon tube. To insulate the SB and SUS vessel, a Teflon cup (yellow-green frame) was placed in the vessel. For gas analysis, V-V, V-4, and V-M were opened and others were closed. For S poisoning tests (SI-4), Si/Ti/Pt substrate was inserted at the Cap along the gas line, where both V-2 and V-3 were opened and V-4 was closed. (b) Time-dependent ion currents for *m/z* = 16 (brown), 18 (light blue), 32 (red), 34 (light green), 44 (black), and 64 (dark green) of an empty SUS vessel for 12 h.

## 4. S poisoning tests

*m/z* = 32 detected in gas analysis can be attributed to either oxygen ($O_2$) or sulfur (S). However, it is impossible to differentiate between these two gases in our system. To investigate the generation of S during charging, we examined sulfur poisoning on platinum (Pt). Silicon (Si) substrates were coated with titanium (Ti, approximately 3 nm thick) and platinum (Pt, approximately 50 nm thick) films using radio frequency (RF) magnetron sputtering. The sputtering conditions are detailed in Table S1. The resulting Si/Ti/Pt substrates (P-sub) were placed at the Cap in the gas line as shown in Figures 1b and S3a. They were exposed to evacuated gases from the SBs totally for 3 hours, that is, at open circuit voltage (OCV: 2.56 V) for 3 hours (OCV-sub) or for 2 hours at OCV followed by 1 hour at 4.55 V (455V-sub). After poisoning, valve-2 (V-2) and valve-3 (V-3) (Figure S3a) were closed, and the gas piping unit between the two valves was transferred into the glove box (GB) to collect the poisoned substrates without exposing them to air. The resultant substrates were then mounted on an XPS holder inside the GB and transported to the XPS using a gas-tight transfer vessel.

XPS measurements were conducted using a PHI5000 system (ULVAC-PHI Inc.) with an AlKα source (1486.6 eV). Both Pt4f and S2p spectra were recorded from a 100 × 100 $\mu m^2$ area, with an energy resolution of 0.2 eV. The XPS data were collected using a radiating neutralizer. The C1s peak position from C–C and C–H bonds, considered as a contamination peak (285.0 eV [3]), was used to normalize the binding energy. In other measurements, which will be described later, XPS was conducted under similar conditions.

Figures S4a and S4b display the Pt4f and S2p XPS spectra measured on the OCV- and 455-subs evacuated from the B-SB, respectively. Both spectra are normalized by the intensity of the Pt4f peak. The spectrum of the P-sub is shown for reference. Two peaks from Pt4f ($Pt4f_{7/2}$ = 71.2 eV and $Pt4f_{5/2}$ = 74.6 eV) did not exhibit an energy shift after the poisoning tests, while the S2p peak appeared from the OCV- and the 455V-subs. Similar results were obtained in the case of the Nb-SB (Figures S4c and S4d). The S/Pt atomic ratio after each preservation was estimated from each peak area in Figures S4a–4d using CASA-XPS software. Figure S4e summarizes the S/Pt atomic ratios from the B-SB and Nb-SB. Although gas analyses detect visible voltage dependencies of *m/z* = 32 at 4.55 V (Figures 3a and 3b), the S/Pt atomic ratio did not show a voltage dependency. Thus, it is reasonable to expect that voltage-dependent *m/z* = 32 is assigned to $O_2$ in our experimental.

We also examined the effects of preservation time on the S/Pt ratios. The Si/Ti/Pt substrates were placed at the Cap in the same manner and exposed them to evacuated gases from the B-SB at OCV for 3, 6, and 15 hours. Figures S4f and S4g show the Pt4f and S2p spectra of the poisoned substrates. Both spectra were normalized by the intensity of the Pt4f peak. The two peaks of Pt4f shifted slightly toward higher binding energies after the poisoning for 6 and 15 hours. [4,5] Figure S4h summarizes the variations in S/Pt atomic ratios with evacuating time. The S/Pt atomic ratio increased from 0.083 after 3 hours to 0.25 after 6 hours. However, the S/Pt atomic ratio after 15 hours (0.24) was almost same as that measured after 6 hours probably because of the saturation of S on Pt surface. These results indicate that S is continuously generated

with preservation time. Thus, we conclude that S is detected in a baseline of *m/z* = 32. Leakage of $H_2O$ from the air to the vessel may provide reactions with the SE and then generate S continuously.

**Table S1**. Deposition conditions of Ti and Pt films by RF sputtering on Si/Ti/Pt substrates.

| Film | thickness | **Deposition conditions** atmosphere, RF power, pressure, T–S distance |
|---|---|---|
| Ti | 3 nm | Ar, 50 W, 4.0 Pa, 45.0 mm |
| Pt | 50 nm | Ar, 40 W, 1.5 Pa, 41.0 mm |

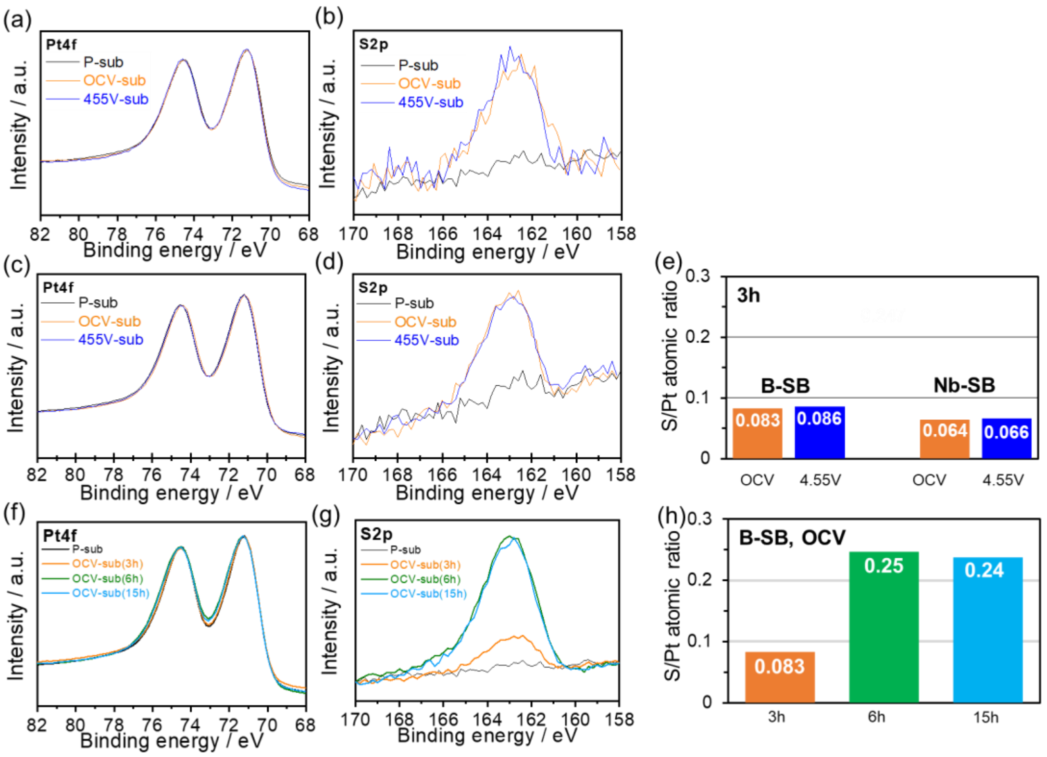


**Figure S4** Pt4f and S2p XPS spectra of the OCV-sub (orange), 455V-sub (blue), and P-sub (black) after S poisoning test of (a, b) the B-SB and (c, d) the Nb-SB. (e) S/Pt atomic ratio estimated from peak area from Figures S4a–4d. XPS spectra of (f) Pt4f and (g) S2p of the OCV-subs after exposing them to the evacuated gases from B-SB for 3h (orange), 6h (green), and 15h (light blue). (h) S/Pt atomic ratio estimated from peak area from Figures S4f and S4g.

## 5. Voltage dependencies of *m/z* (1–100) from charged SBs

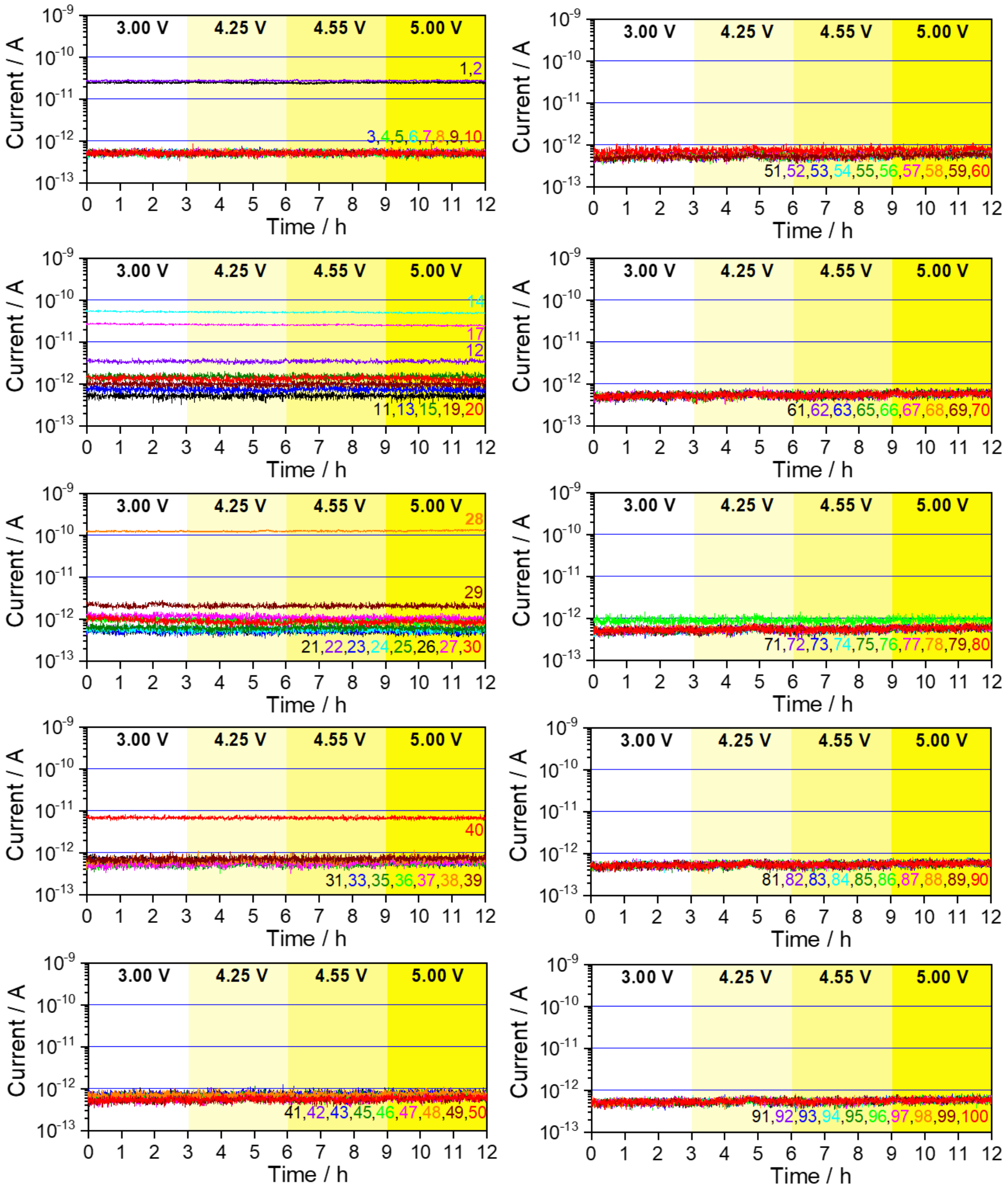


**Figure S5**(a) Time-dependent ion currents for *m/z* =1–100 except for *m/z* = 16, 18, 32, 34, 44, and 64 from charged B-SB at 60ºC.

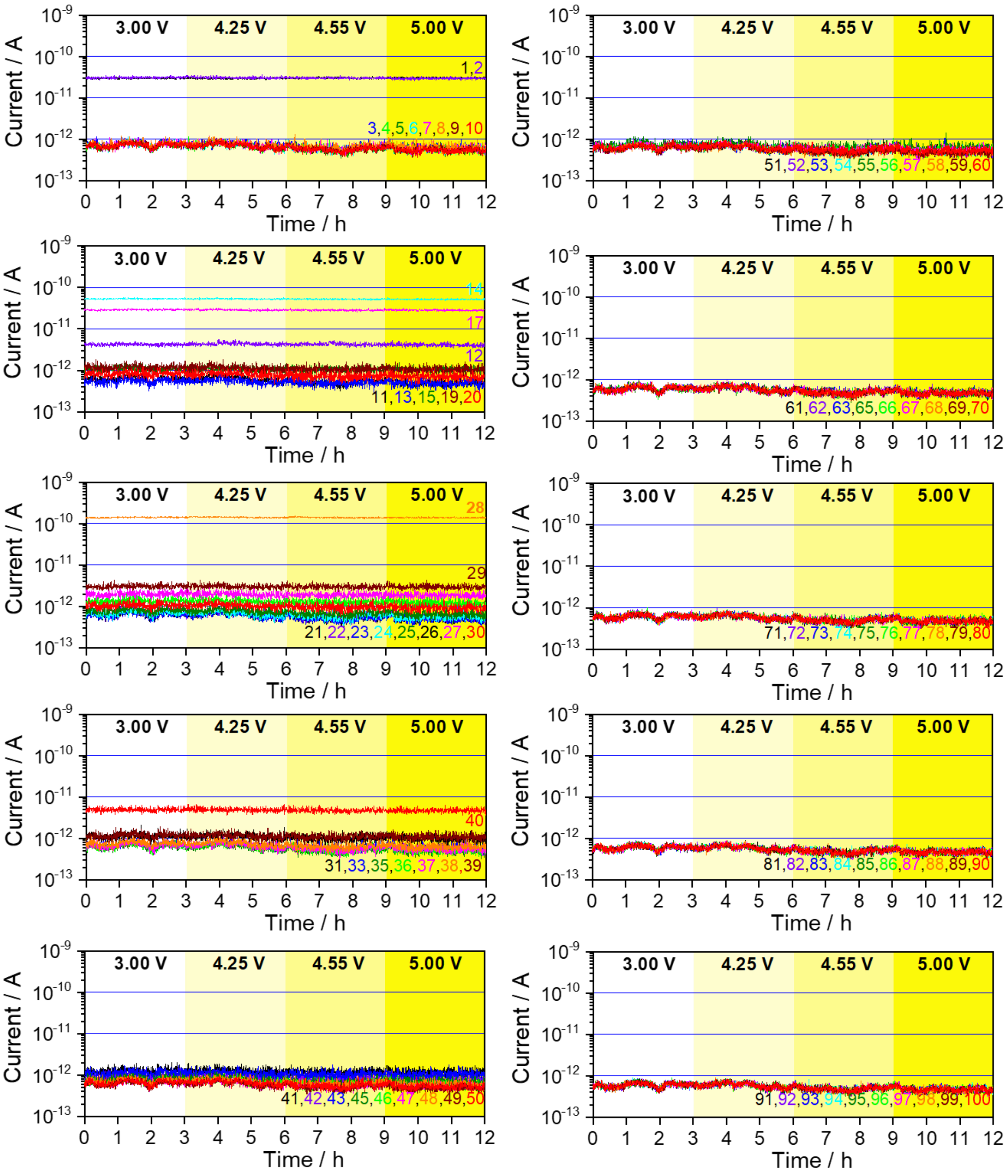


**Figure S5**(b) Time-dependent ion currents for *m/z* =1–100 except for *m/z* = 16, 18, 32, 34, 44, and 64 from charged Nb-SB at 60ºC.

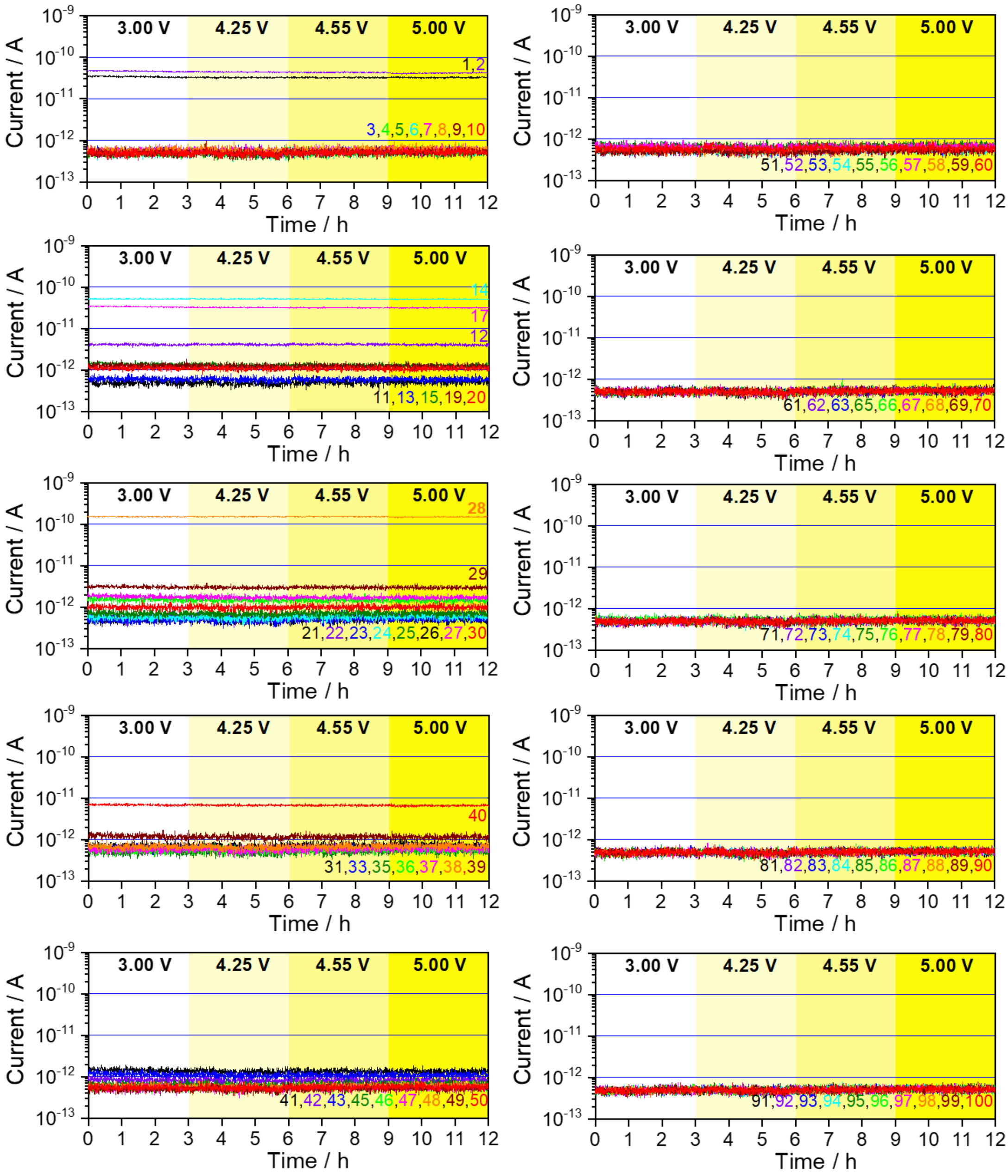


**Figure S5**(c) Time-dependent ion currents for *m/z* =1–100 except for *m/z* = 16, 18, 32, 34, 44, and 64 from charged NbP-SB at 60 ºC.

# 6. Preliminary tests of a-LNbO and a-LNbPO films prepared by PLD as coating layers of SBs

LCO thin film electrodes (80 nm thick) were prepared on platinum (Pt) substrates using PLD. Subsequently, a-LNbO films or a-LNbPO films (30 nm thick) were deposited on the LCO film electrodes. The preparation conditions for those films are summarized in Table S2. All preparations were carried out without exposing the samples to air. The resultant electrodes, both with and without coating layers, were then transferred into an argon-filled glove box using a transfer vessel and subsequently assembled into SBs as illustrated in Figure S6a. In these SBs, both the SE and the anode are LPSCl and an indium-lithium (In–Li) alloy (vs. 0.62 V vs. Li/Li$^+$), respectively, as shown in Figure S1, and the SBs were fabricated by cold pressing at 120 MPa.

Figures S6b–6d display the cyclic voltammetry (CV) curves of the SBs. The voltage was converted to Li/Li$^+$. In the SB using LCO films without coating layers, the peak associated with the redox reactions of LCO was not observed; only irreversible oxidation currents were noted. In contrast, in the SB using LCO films with a 30 nm a-LNbO or a-LNbPO, typical two-phase reaction redox peaks of LCO were observed at 3.9 V. These results indicate that our coating layers prepared by PLD effectively suppress the decomposition of the SE and acts as coating layers for the SB.

**Table S2.** Deposition conditions of LCO, a-LNbO, and a-LNbPO films by PLD for SBs shown in Figure S5a.

| Film | thickness | Target | **Laser conditions**<br>laser type (wavelength), frequency, energy fluence on target | **Deposition conditions**<br>atmosphere, substrate temperature, pressure, T–S distance deposition time | **Annealing conditions**<br>atmosphere, substrate temperature, pressure, time |
|---|---|---|---|---|---|
| LCO | 80 nm | Sintered $Li_{1.4}CoO_2$ (Toshima) | 4th harmonic YAG laser (266 nm), 10 Hz, 0.50 J cm$^{-2}$ | $O_2$, 500 °C, 0.1 Pa, 58.0 mm, 52 min | $O_2$, 500 °C, 10 Pa, 2h |
| a-LNbO | 30 nm | Sintered $LiNbO_3$ (Toshima) | 5th harmonic YAG laser (213 nm), 5 Hz, 0.35 J cm$^{-2}$ | $O_2$, room temperature, 0.05 Pa, 43.0 mm, 60 min | N/A |
| a-LNbPO | 30 nm | Sintered $LiNb_{0.5}P_{0.5}O_3$ (Toshima) | 5th harmonic YAG laser (213 nm), 5 Hz, 0.35 J cm$^{-2}$ | $O_2$, room temperature, 0.05 Pa, 43.0 mm, 60 min | N/A |

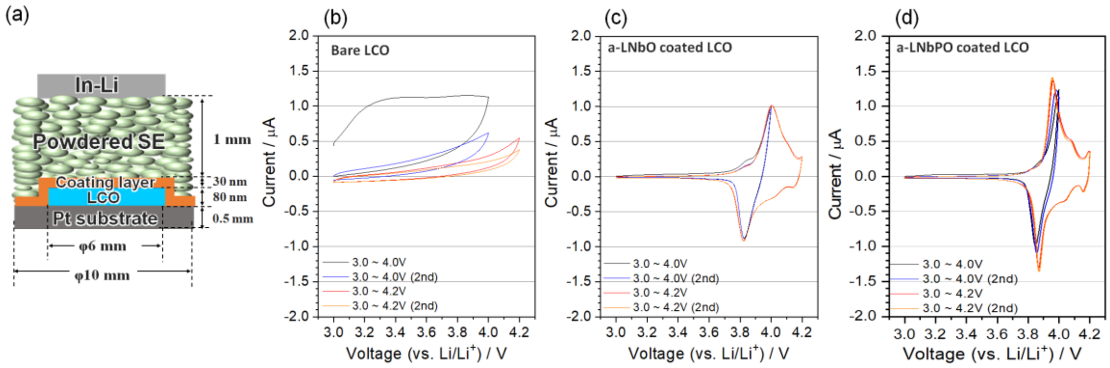


**Figure S6** (a) Schematic image of In–Li/SE/a-LNbO/LCO cell. CVs of the SB at 3.0–4.2 V using (b) bare LCO film, (c) a-LNbO coated LCO film, and (d) a-LNbPO-coated LCO film at 25ºC. $v$ = 0.1 mV s$^{-1}$.

# 7. Operations of model SBs for electrochemical XPS analyses

A model SB in Figure 3a was constructed as follows. A mirror-polished, lithium-ion conductive $Li_2O$–$Al_2O_3$–$TiO_2$–$P_2O_5$–based glass ceramic sheet (LATP sheet, OHARA Inc., 150 µm thick) served as the base solid electrolyte. On one side of the LATP sheet, $LiCoO_2$ (LCO) thin films (30 nm thick) were deposited using PLD and the opposite side, $Fe_2(MoO_4)_3$ (FMO) thin films (300 nm thick) were deposited using PLD. Those preparation conditions are summarized in Table S3. The FMO film was fully coated with platinum (Pt) and gold (Au) films (50 nm thick) as current collectors via RF magnetron sputtering, and the preparations conditions are summarized in Table S4. The capacity of the FMO thin films is approximately 4.5 times higher than that of the LCO film. To employ FMO as a reference and counter electrode in a model SB operating at 3.0 V (vs. $Li/Li^+$),[6] lithium was electrochemically pre-doped into $Li_{0.3}Fe_2(MoO_4)_3$ using dummy LCO films deposited separately adjacent to the LCO film in a model SB. Figure S7a presents the initial charge-discharge curve of the model SB (Type-1), where the LCO thin films were completely covered with Pt current collector films. The voltage was converted to $Li/Li^+$, and typical charge-discharge reactions are observed at 4 V vs. $Li/Li^+$. Additionally, Figure S7b displays the initial charge-discharge curves of another model SB (Type-2), where the Pt current collector film is deposited only around the edge of the LCO film. These two types of SBs demonstrate nearly identical voltage and charge-discharge capacities. Since the electronic conductivity of LCO increases once lithium is extracted from it,[7] Type-2 operates similarly to Type-1. When a-LNbO or a-LNbPO films form on the bare part of the LCO films in Type-2, the model batteries work in the same manner as illustrated in Figure 3b.

**Table S3.** Deposition conditions of LCO and FMO films by PLD for model SBs shown in Figure 3a.

| Film | thickness | Target | **Laser conditions** laser type (wavelength), frequency, energy fluence on target | **Deposition conditions** atmosphere, substrate temperature, pressure, T–S distance, deposition time | **Annealing conditions** atmosphere, substrate temperature, pressure, time |
|---|---|---|---|---|---|
| LCO | 30 nm | Sintered $Li_{1.4}CoO_2$ (Toshima) | 4th harmonic YAG laser (266 nm), 10 Hz, 0.50 J $cm^{-2}$ | $O_2$, 400 °C, 0.1 Pa, 58.5 mm, 36 min | $O_2$, 500 °C, 10 Pa, 1h |
| FMO | 300 nm | Sintered $Fe_2(MoO_4)_3$ (Toshima) | 4th harmonic YAG laser (266 nm), 10 Hz, 1.15 J $cm^{-2}$ | $O_2$, 500 °C, 27 Pa, 46.5 mm, 180 min | N/A |

**Table S4**. Deposition conditions of Pt and Au films by RF sputtering on model SBs.

| Film | thickness | **Deposition conditions** atmosphere, RF power, pressure, T–S distance |
|---|---|---|
| Pt | 50 nm | Ar, 40 W, 1.5 Pa, 41.0 mm |
| Au | 50 nm | Ar, 40 W, 1.5 Pa, 45.0 mm |

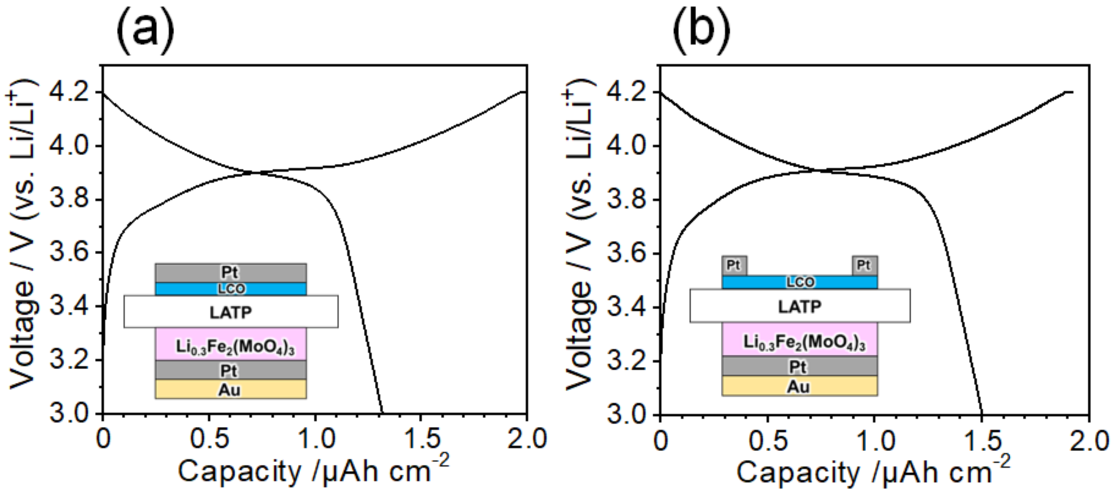


**Figure S7**. The initial charge-discharge curve (3.0–4.2 V vs $Li/Li^+$, $I$ = 1.0 μA $cm^{-2}$, 25ºC) of FMO/LATP/LCO, where (a) current collector film (Pt) was covered entire surface of the LCO and (b) the current collector film (Pt) was covered only the edges of the LCO.

## 8. XPS analyses of an a-LNbO film during charging of a model battery

Electrochemical XPS measurements were performed using the model SB shown in Figure 3a. The SBs were charged to 0.0, 0.5, 0.8, 1.0, 1.2, 1.4 and 1.6 V vs. FMO (3.0, 3.5, 3.8, 4.0, 4.2, 4.4 and 4.6 V (vs. $Li/Li^+$), respectively, using an electrochemical measurement system (SP-300, Bio-logic) at a current density of 1.0 μA $cm^{-1}$ and held at each voltage for 30 min. Then, Li1s, Nb3d, O1s, C1s, and $Co2p_{3/2}$ spectra were measured while maintaining the voltage. Other measurement conditions of XPS are the same as those described in SI-4.

Figure S8a shows the voltage dependency of C1s. The peak around 285 eV is attributed to C–C and C–H bonds, which was used as a reference peak for normalizing the binding energy. The shoulder around 287 eV is assigned to the C–O bond, and the peaks around 289–290 eV to carbonate such as $Li_2CO_3$, respectively. [3,8] Figure S8b shows the voltage dependency of Co2p region, and no peaks were observed at any voltages. This means that pinhole-free a-LNbO films are deposited on LCO films and that only the surface region of the a-LNbO films is detected by XPS analysis. Figure S8c shows the voltage dependency of O1s. The O1s spectra observed at 530.2 eV is attributed to the Nb–O bonding of a-LNbO,[9,10] and the shoulder at 531–533 eV to defect sites with lower coordination[11] or surface carbonate such as $Li_2CO_3$[8] and the C–O bonding.[11] Both peaks basically shifted to higher binding energies with increasing charging voltage above 3.8 V, but the peak has shifted to lower binding energy at 3.5 V. These peak shifts have been observed also for Li1s in Figure 3c. Also, it should be noted that O1s assigned to $Li_2O$ (528.5 eV) was not observed in any voltages,[12] indicating that $Li_2O$ is not separately formed near the surface of a-LNbO at any voltages. Figure S8d shows the voltage dependency of Nb3d. Two peaks derived from $Nb3d_{5/2}$ and $Nb3d_{3/2}$ were observed around 207 eV and 210 eV from higher-valence Nb ($Nb^{4+/5+}$) in amorphous a-LNbO, respectively. [2,9,13,14] At 3.0 V, shoulder peaks originating from lower-valence Nb ($Nb^{3+/4+}$) was observed weakly around 205–206 eV. [13,14] At 3.5 V, the shoulder peak disappeared but the peak shifted to lower binding energy as with O1s and Li1s. Over 3.8 V, both $Nb3d_{5/2}$ and $Nb3d_{3/2}$ peaks shifted to higher binding energy with the charging. [2,15] After discharge to 3.0 V, the Nb3d did not return to original position before charging and shifted further higher binding energy. The XPS peaks from O1s, Nb3d, and Li1s shifted zig-zag directions irregularly against the charge-discharge voltages even the binding energy was normalized by C1s peak in each voltage. These peak shifts may contain the effects of charge-up inside the coating layers at a given voltage. For example, when C1s, O1s, and Nb3d spectra were measured at 3.0 V with increasing the neutralizer current from 3 μA or 15 μA, both O1s and Nb3d shifted to lower binding energy though the peaks were normalized by C1s (surface) peak. Thus, it is difficult to discuss on the oxidation states of the a-LNbO film at each charged or discharged voltage from the binding energy shift of XPS spectra. This situation was same in the case of a-LNbPO film as will be discussed in SI-10.

To investigate Li distribution inside the a-LNbO film after charge-discharge reactions, depth profiles were measured. The a-LNbO film was etched for 10 min using an Ar ion (acceleration voltage: 2 kV, emission current: 7 mA, irradiation area: 2 × 2 $mm^2$), and both Li1s and $Co2p_{3/2}$ spectra were measured

after every 1 min etching. After etching, C1s peak was almost disappeared and then peak positions were not normalized by C1s. The data was measured with radiating neutralizer. Figure S8e summarizes the $Co2p_{3/2}$ spectra during the etching for 5–10 min. The $Co2p_{3/2}$ peak appeared after the etching for 7 min, and the peak appeared clearly by further etching. Figure S8f summarizes Li1s spectra during the etching for 5–10 min. Li1s was not observed until the etching of 8 min, and a weak peak appeared after 9 min etching. Since the $Co2p_{3/2}$ peak was observed after the etching for 7 min, the Li1s observed after 9 min etching is attributed to LCO, not a-LNbO. These results indicate that Li inside 30 nm-thick a-LNbO film is almost extracted after charge-discharge reactions and that Li is not accumulated around the LCO/a-LNbO interface. Because $Co2p_{3/2}$ is observed before the appearance of Li1s peak after 7–8 min etching, $Co^{2+}$ may diffuse to a-LNbO thin film after charge-discharge reactions, a phenomenon previously reported.[16]

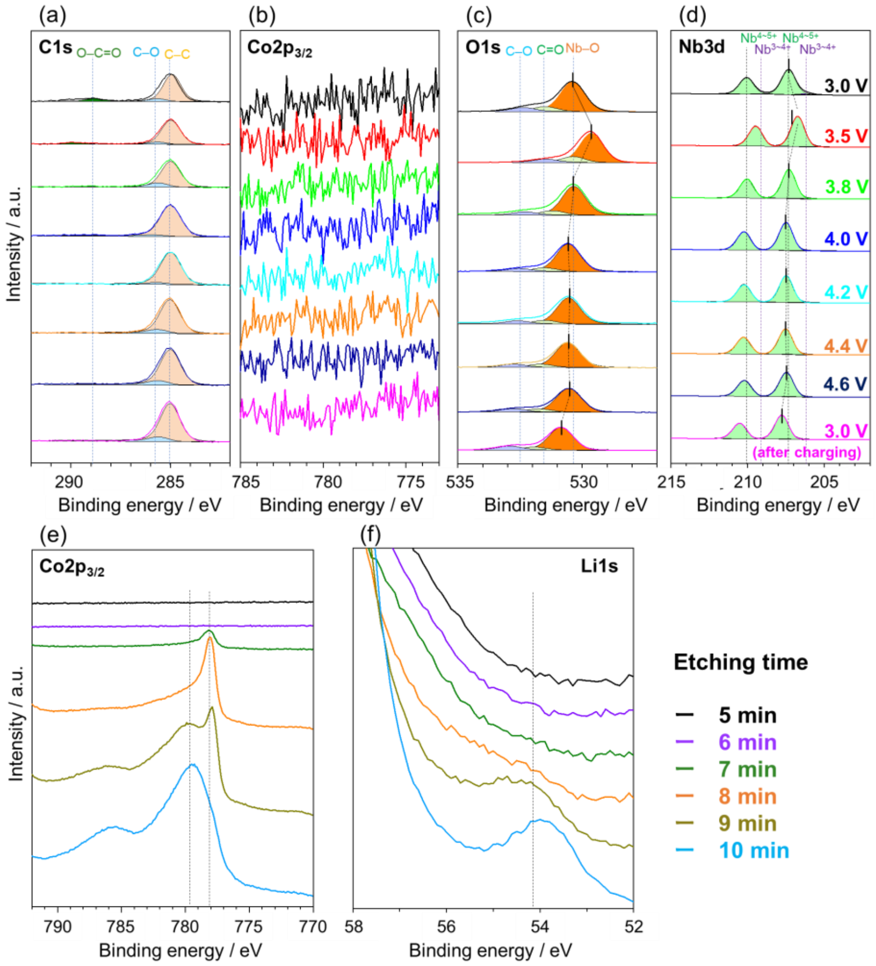


**Figure S8**. Voltage dependencies of the XPS spectra of (a) C1s, (b)$Co2p_{3/2}$, (c) O1s, and (d) Nb3d at 3.0 V (black), 3.5 V(red), 3.8 V(light green), 4.0 V(blue), 4.2 V(light blue), 4.4 V(orange), 4.6 V(dark blue), and 3.0 V after charging (pink). Depth XPS profiles inside the a-LNbO film of (e) $Co2p_{3/2}$ and (f) Li1s after discharging at 3.0 V. Etching time was 5 min. (black), 6min. (purple), 7 min. (green), 8 min. (orange), 9 min. (light brown), and 10 min. (light blue).

## 9. Electronic Properties of a-LNbO and a-LNbPO films prepared by PLD

Ultraviolet photoelectron spectroscopy (UPS) and low-energy inverse photoelectron spectroscopy (LEIPS) measurements were conducted to evaluate the electronic properties of a-LNbO films and a-LNbPO films.[17] Both a-LNbO films and a-LNbPO films (SI-6) were prepared on Si/Ti/Pt substrates (SI-4). The UPS measurements utilized a He1 light source, and the energy resolution was 0.01 eV. LEIPS measurements were conducted by supplying an electron beam with an emission current of $1.0\times10^{-6}$ A, achieving an energy resolution of 0.04 eV. Figure S9a displays the UPS and LEIPS profiles of these films. Both the valence band maximum (VBM) and the conduction band minimum (CBM) energies were estimated from an inflection point of the UPS-LEIPS slopes, which is determined by second-order differential of each slope in the displayed scale. Figure S9b summarizes the schematic image of band structures of these films, specifically their VBM, CBM, and Fermi energy ($E_F$). In these films, the values of VBM are same (5.8 eV) and the values of $E_F$ are also almost same (4.1–4.2 eV). Therefore, it is expected that anodic potential window is almost same in these two films. Cathodic potential window of a-LNbPO may be slightly lower than that of a-LNbO because the CBM of a-LNbPO (3.2 eV) is lower than that of a-LNbO (3.6 eV), though we have not checked it by electrochemical analysis.

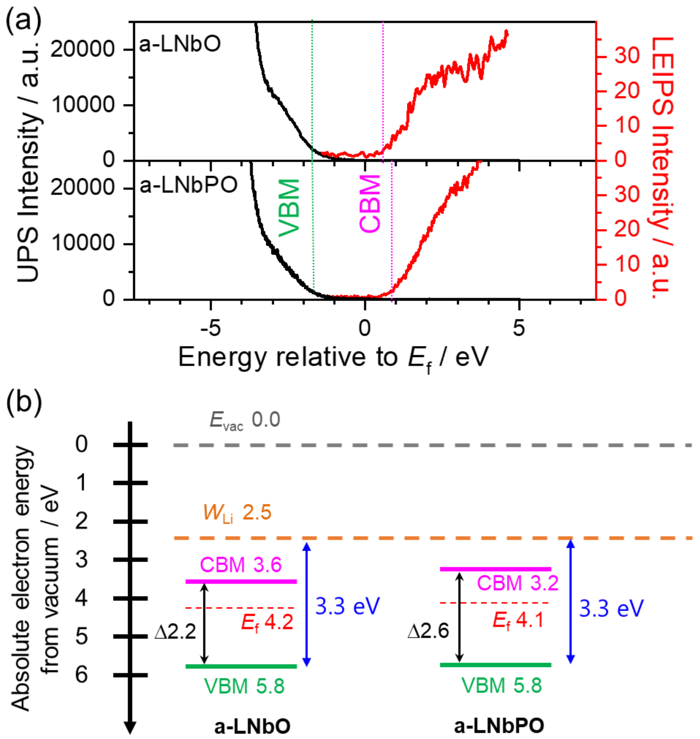


**Figure S9**. (a) UPS-LEIPS profiles of the a-LNbO and a-LNbPO prepared on Si/Ti/Pt substrates. (b) Schematic band structure of a-LNbO and a-LNbPO estimated from Figure S9a. Values of the work functions of Li ($W_{Li}$, orange [18]), VBM (green), CBM (pink), and Fermi level ($E_f$, red) are all against the vacuum level ($E_{vac}$, gray).

## 10. XPS analyses of an a-LNbPO film at charging of a model battery

Electrochemical XPS measurements were conducted on a model solid battery (SB) using a-LNbPO (P/Nb~0.5) in place of a-LNbO. At 3.0 V (initial state), the Li/(Nb + P) and O/(Nb + P) ratios were 0.55 and 2.73, respectively. Assuming that Nb is pentavalent and is 6-coordinbate by O and P is pentavalent and is 4-coordinate by O, the ideal O/(Nb+P) ratio will be 3 at sufficiently polymerized state. Thus, O/(Nb + P) ratio lower than 3 suggests that further O deficient bonding state is realized in a-LNbPO film, though details of this polymerized structure is not clarified in this work and is currently investigated. Potential dependence of Li1s peak and Li/(Nb + P) and O/(Nb + P) atomic ratios are shown in Figure 3e and 3f, respectively. Figures S10a–e display potential dependences of C1s, Co2p$_{3/2}$, O1s, Nb3d, and P1s XPS spectra of the a-LNbPO film, respectively. As mentioned in S8, the binding energy changed zig-zag direction irregularly depending on the voltage probably because of charge-up of the a-LNbPO film, though all the spectra were normalized by C1s peak. Then, it is difficult to discuss on the oxidation states of each element from these spectra. O1s assigned to $Li_2O$ (528.5 eV) was not observed at any voltages as with a-LNbO (Figure S8c), but it is interesting to note that O1s spectra are visibly separated into two peaks during the charging at high voltages as shown by dotted lines in Fig. S10c. We predict that this shoulder peak is assinded to peroxide-like $O^-$ formation.[19] Assuming that both Nb and P are pentavalent and both [$NbO_6$] octahedra and [$PO_4$] tetrahedra units are connected only by corner-sharing, ideal O/(Nb + P) ratio is 3 which is close to expeimental value (2.73). At the charging process, a-LNbPO may oxidize lattice-$O^{2-}$ to $O^-$, and $O_2$ generation may be supressed probably because most of lattice-Os will be strongly bonded with P. Because O/(Nb + P) ratio has slightly increased over 4.4 V, uncaptured oxygen may be trapped by breaking corner-sharing bonding in part. Of course, further analyses are required to clarify the above-mentioned mechanism in detail to confirm microscopic guideline to develop advanced coating materials for high energy density SBs.

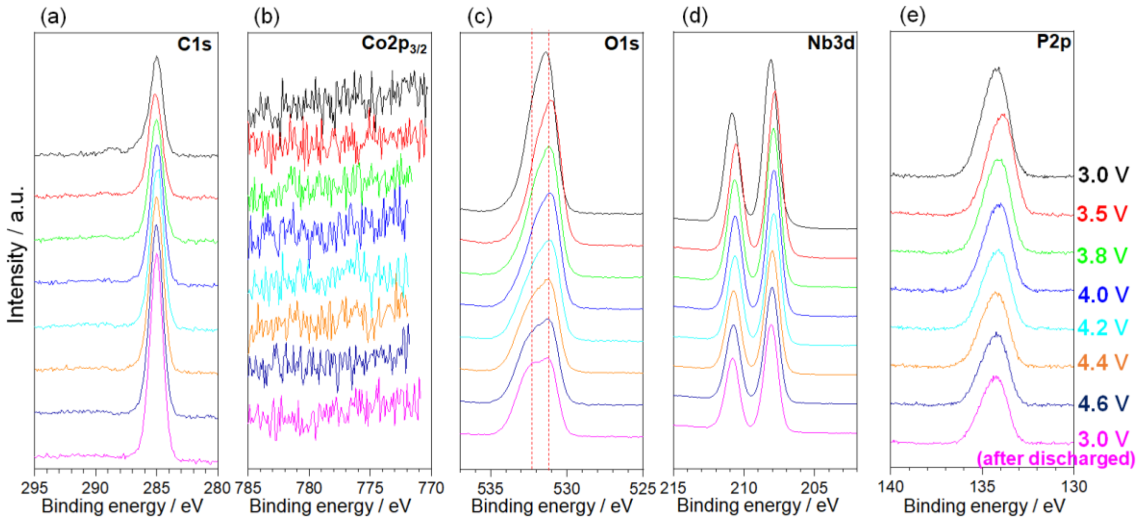


**Figure S10.** Voltage dependencies of the XPS spectra of (a) C1s, (b)Co2p$_{3/2}$, (c) O1s, (d) Nb3d, and (e) P2p on a-LNbPO at 3.0 V (black), 3.5 V(red), 3.8 V(light green), 4.0 V(blue), 4.2 V(light blue), 4.4 V(orange), 4.6 V(dark blue), and 3.0 V after discharge (pink).

## 11. References


[1] Y. Morino, S. Kanada, *J. Power Sources* **2021**, *509*, 230376.

[2] Y. Morino, A. Shiota, S. Kanada, W. S. K. Bong, K. Kawamoto, Y. Inda, H. Tsukasaki, S. Mori, Y. Iriyama, *ACS Appl. Mater. Interfaces* **2023**,*15*, 36086−36095.

[3] D. Briggs, and G. Beamson, *Anal. Chem.* **1992**, *64* (15), 1729–1736.

[4] R. Ahmadi and M. K. Amini, *Int. J. Hydrogen Energy* **2011**, *36*, 7275–7283.

[5] C. Dablemont, P. Lang, C. Mangeney, J. Y. Piquemal, V. Petkov, F. Herbst, G. Viau, *Langmuir* **2008**, *24*, 5832–5841.

[6] Y. Kee, Y. Suzuki, N. Ishigaki, M. Motoyama, Y. Kimura, K. Amezawa, Y. Iriyama, *Electrochem. Commun.* **2021**, *130*, 107108.

[7] M. Shibuya, T. Nishina, T. Matsue, I. Uchida, *J. Electrochem. Soc.* **1996**, *143*, 3157–3160.

[8] X. Rao, Y. Lou, J. Zhao, J. Chen, Y. Qiu, T. Wu, S. Zhong, H. Wang, L. Wu, *J. Porous Mater.* **2023**, *30* (2), 403– 419.

[9] N. Kaufherr, D. J. Eichorst, D. A. Payne, *J. Vac. Sci. Technol., A* **1996**, 14, 299–306.

[10] E. A. Skryleva, I. V. Kubasov, P. V. Kiryukhantsev-Korneev, B. R. Senatulin, R. N. Zhukov, K. V. Zakutailov, M. D. Malinkovich, Y. N. Parkhomenko, *Appl. Surf. Sci.* **2016**, *389*, 387–394.

[11] P. Jing, K. Liu, L. Soule, J. Wang, T. Li, B. Zhao, M. Liu, *Nano Energy* **2021**, *89*, 106398.

[12] K. P. C. Yao, D. G. Kwabi, R. A. Quinlan, A. N. Mansour, A. Grimaud, Y.-L. Lee, Y.-C. Lu, Y. Shao-Horn, *J. Electrochem. Soc.* **2013**, *160*, A824.

[13] F. Chen, W. P. Chen, Y. Wang, Y. M. Hu, Z. J. Shen, H. L. W. Chan, *Physica B* **2011**, *406*, 683–686.

[14] K. Tabata, M. Kamada, T. Choso, H. Munakata, *Appl. Surf. Sci.***1998**, *125*, 93−98.

[15] H. Zhai, H. Liu, H. Li, L. Zheng, C. Hu, Z. Wang, J. Qi, J. Yang, *Nanoscale Res. Lett.* **2017**, *12*, 496.

[16] A. Sakuda, A. Hayashi, M. Tatsumisago, *Chem. Mater.* **2010**, *22*, 949–956.

[17] F. Nakayama, Y. Suzuki, K. Yoshikawa, S. Yamamoto, M. Sakakura, T. Ohnishi, Y. Iriyama, *Chem. Commun.* **2022**, *58*, 13262−13265.

[18] A. Etxebarria, S. L. Koch, O. Bondarchuk, S. Passerini, G. Teobaldi, M. Á. Muñoz-Márquez, *Adv. Energy Mater.* **2020**, *10*, 2000520.

[19] K. Shimoda, T. Minato, K. Nakanishi, H. Komatsu, T. Matsunaga, H. Tanida, H. Arai, Y. Ukyo, Y. Uchimoto, Z. Ogumi, J. Mater. Chem. A. 2016, 4, 5909−5916.
